# Use of Transcarotid Artery Revascularization, Transfemoral Carotid Artery Stenting, and Carotid Endarterectomy in the US From 2015 to 2019

**DOI:** 10.1001/jamanetworkopen.2022.31944

**Published:** 2022-09-16

**Authors:** David P. Stonko, Earl Goldsborough, Pavel Kibrik, George Zhang, Courtenay M. Holscher, Caitlin W. Hicks

**Affiliations:** 1 Department of Surgery, The Johns Hopkins Hospital, Baltimore, Maryland; 2 R. Adams Cowley Shock Trauma Center, University of Maryland, Baltimore; 3 Division of Vascular Surgery and Endovascular Therapy, Department of Surgery, The Johns Hopkins Hospital, Baltimore, Maryland; 4 Vascular Institute of New York, Brooklyn, New York; 5 Department of Surgery, Brigham and Women’s Hospital, Boston, Massachusetts

## Abstract

**Importance:**

A transcarotid artery revascularization (TCAR) device was approved by the US Food and Drug Administration in 2015 for carotid revascularization in patients at high risk for stroke, cranial nerve injury, or major cardiac event. It is unclear how the introduction of TCAR has changed the use of carotid endarterectomy (CEA) and transfemoral carotid artery stenting (TFCAS).

**Objective:**

To quantify the temporal changes in the operative approach to carotid revascularization (CEA vs TFCAS vs TCAR), and to identify patient and disease characteristics commonly associated with each approach.

**Design, Setting, and Participants:**

This retrospective cohort study obtained data from the Vascular Quality Initiative database from January 1, 2015, to December 31, 2019. Patients with carotid artery stenosis who underwent CEA, TFCAS, or TCAR were included. Data were analyzed from January to April 2022.

**Exposures:**

Month and year of surgery as well as patient risk status.

**Main Outcomes and Measures:**

Number and proportion of carotid revascularization procedures by operative approach.

**Results:**

A total of 108 676 patients (mean [SD] age 56.6 [12.5] years; 66 684 men [61.4%]) were included in the analysis. The most common operative approach overall was CEA (n = 81 508 [75.0%]), followed by TFCAS (n = 15 578 [14.3%]) and TCAR (n = 11 590 [10.7%]). The number of procedures increased over the study period (16 754 in 2015 vs 27 269 in 2019; *P* < .001). In 2015, CEA was used in 84.9% of all cases, followed by TFCAS (14.4%) and TCAR (0.8%). In 2019, CEA was used in 64.8% of cases, followed by TCAR (21.9%) and TFCAS (13.3%). The proportional use of CEA decreased by 5.0% (95% CI, −7.4% to −2.6%) per year, and TCAR use increased by 5.3% (95% CI, 2.3%-8.3%) per year. Among patients at high risk, the change was greater: CEA use decreased by 7.8% (95% CI, −11.9% to −3.8%) per year, TFCAS decreased by 4.8% (95% CI, −9.5% to −0.14%) per year, and TCAR increased by 12.6% (95% CI, 7.1%-18.1%) per year. Multinomial logistic regression showed that patient risk status was the most important characteristic associated with TCAR compared with CEA (relative risk ratio, 36.10; 95% CI, 29.24-44.66; *P* < .001) and TFCAS (relative risk ratio, 14.10; 95% CI, 11.86-16.66; *P* < .001). Linear regression revealed no association between year of surgery and in-hospital myocardial infarction, stroke, or mortality.

**Conclusions and Relevance:**

Results of this study indicate that TCAR has become the dominant carotid revascularization approach, surpassing TFCAS and CEA in patients at high risk for stroke, cranial nerve injury, or cardiovascular events. Patient high-risk status was the main characteristic associated with a stenting approach, highlighting the perceived importance of carotid stenting therapies in this patient population.

## Key Points

**Question:**

How has the use of transcarotid artery revascularization (TCAR) changed since the US Food and Drug Administration approved the first TCAR device in 2015 compared with the change in use of carotid endarterectomy (CEA) and transfemoral carotid artery stenting (TFCAS) from 2015 to 2019?

**Findings:**

In this cohort study of 108 676 patients who underwent carotid revascularization, TCAR was used predominantly in 53.2% of patients at high risk and was the most common stenting modality in 21.9% of all patients; CEA use decreased by 5.0% per year and TCAR use increased by 5.3% per year.

**Meaning:**

Findings of this study suggest that TCAR use has become the primary carotid revascularization approach in the US, surpassing TFCAS and CEA, among patients at high risk for stroke, cranial nerve injury, or cardiovascular events.

## Teaser

This cohort study examines the pattern of use, outcomes, and patient and disease factors associated with transcarotid artery revascularization (TCAR), transfemoral carotid artery stenting (TFCAS), and carotid endarterectomy (CEA) in the US from 2015 to 2019.

## Introduction

Carotid endarterectomy (CEA) has historically been considered the standard operative therapy for carotid artery stenosis for both symptomatic^1,2,3^ and asymptomatic patients.^4,5^ As best medical therapy has evolved, however, the role of carotid revascularization in asymptomatic patients has become less clear.^6,7,8^

In 2004, Yadav et al^9^ published the results of the SAPPHIRE (Stenting and Angioplasty With Protection in Patients at High Risk for Endarterectomy) randomized clinical trial, which established that transfemoral carotid artery stenting (TFCAS) with an embolic protection device was not inferior to CEA among patients with severe carotid artery stenosis and coexisting conditions, and continued safety was preserved in long-term follow-up.^10^ Although the evolution of best medical therapy was associated with decreasing overall rates in carotid revascularization between 2000 and 2016, TFCAS as a proportion of overall revascularization rates increased since the SAPPHIRE trial was published in 2004.^11^ In 2015, Kwolek et al^12^ published the results of the ROADSTER (Safety and Efficacy Study for Reverse Flow Used During Carotid Artery Stenting Procedure) multicenter trial, which established that transcarotid artery revascularization (TCAR) was also safe and effective, with a low overall stroke rate at 30 days (1.4%) and durable outcomes at 1-year postoperative follow-up.^13,14^ These results led to the first US Food and Drug Administration (FDA)–approved TCAR device.^15^ Since this approval, there has been a rapid increase in TCAR adoption,^7^ particularly given that contemporary data from the Vascular Quality Initiative (VQI) have shown excellent results.^16,17,18,19^

The goal of this cohort study was to quantify the temporal changes in the operative approach to carotid revascularization (CEA vs TFCAS vs TCAR) since the approval of the first TCAR device, and to identify patient and disease characteristics commonly associated with each approach. A secondary aim was to evaluate the association of outcomes with year of surgery, with additional subanalyses in high-volume vs low-volume TCAR centers.

## Methods

### Study Population

Using the VQI database, we identified patients with carotid artery stenosis who underwent CEA, TFCAS, or TCAR from January 1, 2015, through December 31, 2019. Institutional review board approval was obtained from The Johns Hopkins University School of Medicine, which waived the patient informed consent requirement because this study was a retrospective analysis of publicly available deidentified data. We followed the Strengthening the Reporting of Observational Studies in Epidemiology (STROBE) reporting guideline.^20^

At the time of study design, VQI data were available through September 2020. Because we aimed to evaluate the temporal changes of carotid revascularization in the context of disease and patient characteristics, we truncated the analysis period to before the COVID-19 pandemic. Patients with missing baseline characteristics (n = 488) or operative approach (n = 418) were excluded. Patients with unknown or less than 50% stenosis (n = 4946) or 100% stenosis (n = 2124) were also excluded.

### Definitions

The patient comorbidities reported were defined by the VQI. Moderate-grade stenosis was defined as 50% to 79% stenosis, and high-grade stenosis was defined as 80% or greater stenosis. Patients were classified as being symptomatic if they had a recorded history of amaurosis fugax, transient ischemic attack, or stroke within 6 months of undergoing carotid revascularization. Patients were deemed as high risk for CEA according to the high-risk physiologic or anatomic criteria within the VQI database and the Centers for Medicare & Medicaid Services guidelines^21^ (ie, myocardial infarction [MI] within past 6 months, unstable angina, congestive heart failure class III or IV, creatinine level >2.5 mg/dL [to convert to micromoles per liter, multiply by 76.25], previous ipsilateral CEA, or radiotherapy).

### Evaluation of Temporal Changes 

The primary outcomes of this analysis were the number and proportion of carotid revascularizations by operative approach (CEA, TFCAS, or TCAR). Patients were grouped by operative approach and analyzed by the month and year of surgery. Changes in monthly and annual case volumes were captured graphically using heat maps and scatterplots. Because different institutions participate in the VQI each year, the data cannot be considered to be a true cross-sectional representation of the national patterns overall. Therefore, the proportions of all carotid revascularization procedures by operative approach were evaluated. These proportions were captured graphically using stacked bar charts and scatterplots with linear splines for all included patients and for those in the high-risk category alone. Overall yearly temporal changes in carotid revascularization approach were then compared using a nonparametric Wilcoxon-type test for 3 independent groups (Cuzick test), and individual years were compared using χ^2^ statistics. A similar approach was used to explore temporal changes in a subgroup of patients at high risk for stroke, cranial nerve injury, or cardiovascular events as a sensitivity analysis.

### Statistical Analysis

Patient characteristics (age, self-reported race and ethnicity [which were reported by the centers and included American Indian or Alaska Native, Asian, Black, Hispanic, Native Hawaiian or Other Pacific Islander, White, more than 1 race, and unknown or other]), sex, insurance payer, functional status, smoking status, and comorbidities) and carotid disease characteristics (degree of stenosis and symptomatic status) were divided by operative approach (CEA, TFCAS, or TCAR) and compared using χ^2^ statistics. Multinomial logistic regression was used to identify the independent patient and disease characteristics associated with each approach. These characteristics are reported as relative risk ratios (RRRs) for an approach given 3 possible approaches, with CEA considered to be the reference. We identified covariates for the multinomial model a priori, and after confirming no collinearity, we included the following covariates in the multivariable model: age, sex, race and ethnicity, insurance status, comorbidities (hypertension, coronary artery disease, congestive heart failure, chronic obstructive pulmonary disease, diabetes, and chronic kidney disease [CKD] or hemodialysis), functional status (fully functional or not fully functional), smoking status (never, previous, current), high-risk vs standard-risk status, degree of stenosis (moderate grade or high grade), symptomatic status, and year of surgery.

We created clusters by hospital to account for possible hospital-based practice variation. We then performed a sensitivity analysis including only patients who underwent carotid revascularization procedures in 2019 to capture the contemporary state of therapy choice to assess whether associations existed immediately after FDA approval of TCAR. Covariates included in the sensitivity analysis were the same as those used in the main multivariable model with the exception of year of surgery, which was removed because all of these procedures were performed in 2019.

Additional analysis was performed to compare overall in-hospital outcomes of carotid revascularization (stroke, MI rate, and mortality) over time, and these outcomes were then stratified by TCAR volume status. In the overall analysis, simple linear regression with computation of 95% CIs was performed to compare these outcomes during the study period, and the *F* test was used to test a slope that was not equal to 0. To ascertain whether a center was designated as high or low volume, a frequency analysis was performed that tallied the number of TCARs that each center performed from 2015 to 2019. A histogram and a violin plot were used to represent these data graphically. Centers that performed more than 40 TCARs from 2015 to 2019 were identified as high volume. In-hospital outcomes for high-volume and low-volume TCAR centers were then compared over time. Analyses consisted of testing the association between year of surgery, TCAR volume status, and in-hospital outcome using unpaired, 2-tailed *t* tests for multiple comparisons. We also compared in-hospital complications directly using *t* tests without including year of surgery.

A 2-sided *P* < .05 was considered to be significant for all analyses. Statistical analysis was performed with Stata, version 17 (StataCorp LLC), and figures were created using GraphPad Prism, version 9.3.0 (GraphPad Software). Data were analyzed from January to April 2022.

## Results

There were 108 676 patients included in the analysis, of whom 41 992 were women (38.6%) and 66 684 were men (61.4%) with a mean (SD) age of 56.6 (12.5) years. The most common carotid revascularization approach overall was CEA (n = 81 508 [75.0%]), followed by TFCAS (n = 15 578 [14.3%]) and TCAR (n = 11 590 [10.7%]). Patient demographic, comorbidity, and disease characteristics by operative approach are provided in Table 1. Median (IQR) postoperative length of stay was 1 (1-2) day for TFCAS, 1 (1-2) day for TCAR, and 1 (1-24) day for CEA.

**Table 1.** Baseline Patient Demographic, Comorbidity, and Disease Characteristics of Patients in the VQI Database Who Underwent Carotid Revascularization, From 2015 to 2019

Characteristic	No. (%)			*P* value
	CEA (n = 81 508)	TCAR (n = 11 590)	TFCAS (n = 15 578)	
Total No. of patients	108 676			
Age, mean (SD), y	51.71 (9.05)	73.06 (9.06)	68.12 (10.43)	<.001
Sex				
Female	32 173 (39.5)	4311 (37.2)	5508 (35.4)	<.001
Male	49 335 (60.5)	7279 (62.8)	10 070 (64.6)	<.001
Race and ethnicity^a^				
Black	3798 (4.7)	522 (4.5)	913 (5.9)	
White	73 745 (90.5)	10 503 (90.6)	13 951 (89.6)	
Other^b^	3938 (4.8)	559 (4.8)	711 (4.6)	<.001
Insurance payer				
Medicare	46 123 (56.6)	7626 (65.8)	8815 (56.6)	
Medicaid	3052 (3.7)	331 (2.9)	761 (4.9)	
Commercial	20 410 (25.0)	3274 (28.3)	5424 (34.8)	
Other or self-pay^c^	1877 (2.3)	320 (2.8)	563 (3.6)	<.001
Smoking status				
Never	20 836 (25.6)	2973 (25.7)	3944 (25.3)	
Previous	39 917 (49.0)	5987 (51.7)	7313 (46.9)	
Current	20 724 (25.4)	2618 (22.6)	4295 (27.6)	<.001
Functional status				
Fully	72 398 (88.8)	7608 (65.6)	11 051 (70.9)	
Not fully	8982 (11.0)	3775 (32.6)	4405 (28.3)	<.001
Comorbidities				
Hypertension	72 840 (89.4)	10 542 (91.0)	13 624 (87.5)	<.001
Diabetes	29 852 (36.6)	4385 (37.8)	5943 (38.2)	<.001
CAD	22 109 (27.1)	6113 (52.7)	9952 (63.9)	<.001
CHF	9306 (11.4)	2024 (17.5)	2544 (16.3)	<.001
COPD	18 984 (23.3)	3162 (27.3)	4180 (26.8)	<.001
CKD or HD	1050 (1.3)	201 (1.7)	246 (1.6)	<.001
Symptomatic status	38 881 (47.7)	4496 (38.8)	6966 (44.7)	<.001
Elective	71 502 (87.7)	10 405 (89.8)	12 134 (77.9)	
Urgent or emergent	9982 (12.3)	1183 (10.2)	3426 (22.0)	<.001
Degree of stenosis				
Moderate: 50%-79%	33 231 (40.8)	3721 (32.1)	5430 (34.9)	
Severe: 80%-99%	48 277 (59.2)	7869 (67.9)	10 148 (65.1)	<.001
Risk category				
Standard	71 519 (87.7)	2051 (17.7)	5014 (32.2)	
High	9989 (12.3)	9539 (82.3)	10 564 (67.8)	<.001

Abbreviations: CAD, coronary artery disease; CEA, carotid endarterectomy; CHF, congestive heart failure; COPD, chronic obstructive pulmonary disease; CKD, chronic kidney disease; HD, hemodialysis; TCAR, transcarotid artery revascularization; TFCAS, transfemoral carotid artery stenting; VQI, Vascular Quality Initiative.

^a^ Race and ethnicity were reported in the VQI database. Not all races and ethnicities in the registry were included because of the small number of people in each group.

^b^ Other race and ethnicity groups were American Indian or Alaska Native, Asian, Hispanic, Native Hawaiian or Other Pacific Islander, more than 1 race, and unknown or other.

^c^ Other insurance payers were those that were not Medicare, Medicaid, commercial, or self-pay as coded in the VQI.

### Temporal Changes in All Operative Approaches

**Figure 1.**
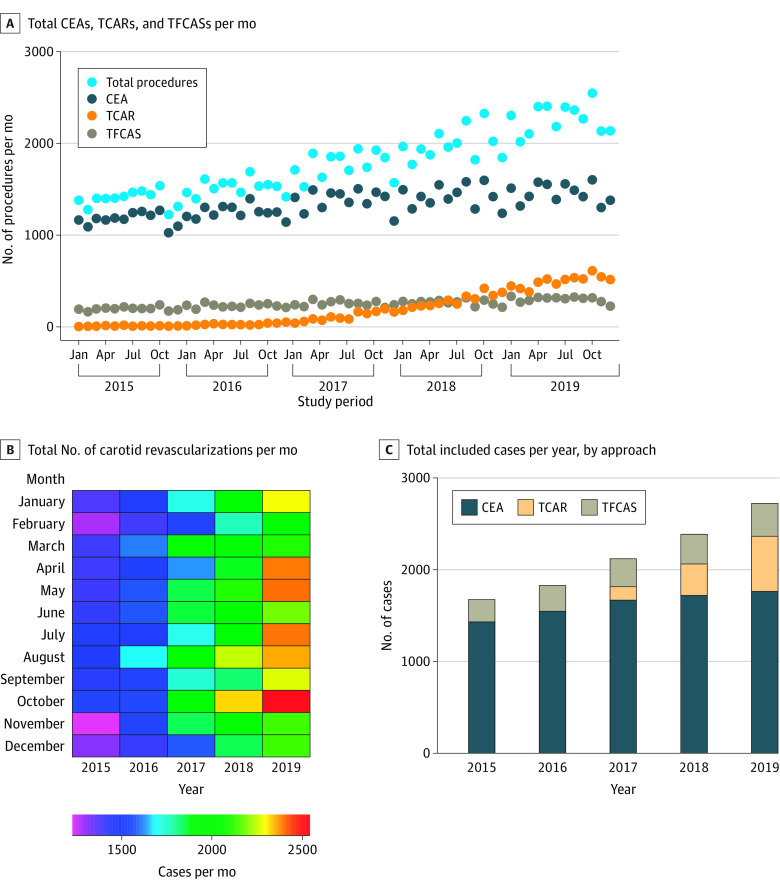
Carotid Revascularization Procedures per Month in the Vascular Quality Initiative, From January 2015 to December 2019 CEA indicates carotid endarterectomy; TCAR, transcarotid artery revascularization; TFCAS, transfemoral carotid artery stenting.

The total number of carotid revascularization procedures included in the VQI database per year increased significantly over the study period (16 754 in 2015 vs 27 269 in 2019; *P* < .001) (Figure 1A) but with minimal month-to-month variation in total case numbers (Figure 1B). This variation represents a 13.0% (95% CI, 10.3%-15.7%) per year increase in the total number of procedures during the study period. Use of individual operative approaches over time varied significantly. The number of CEA (14 219 in 2015 vs 17 664 in 2019; *P* < .001) and TFCAS (2405 in 2015 vs 3633 in 2019; *P* < .001) procedures performed per year increased slightly, whereas the number of TCAR procedures performed per year increased substantially (130 in 2015 vs 5972 in 2019; *P* < .001) (Figure 1C).

**Figure 2.**
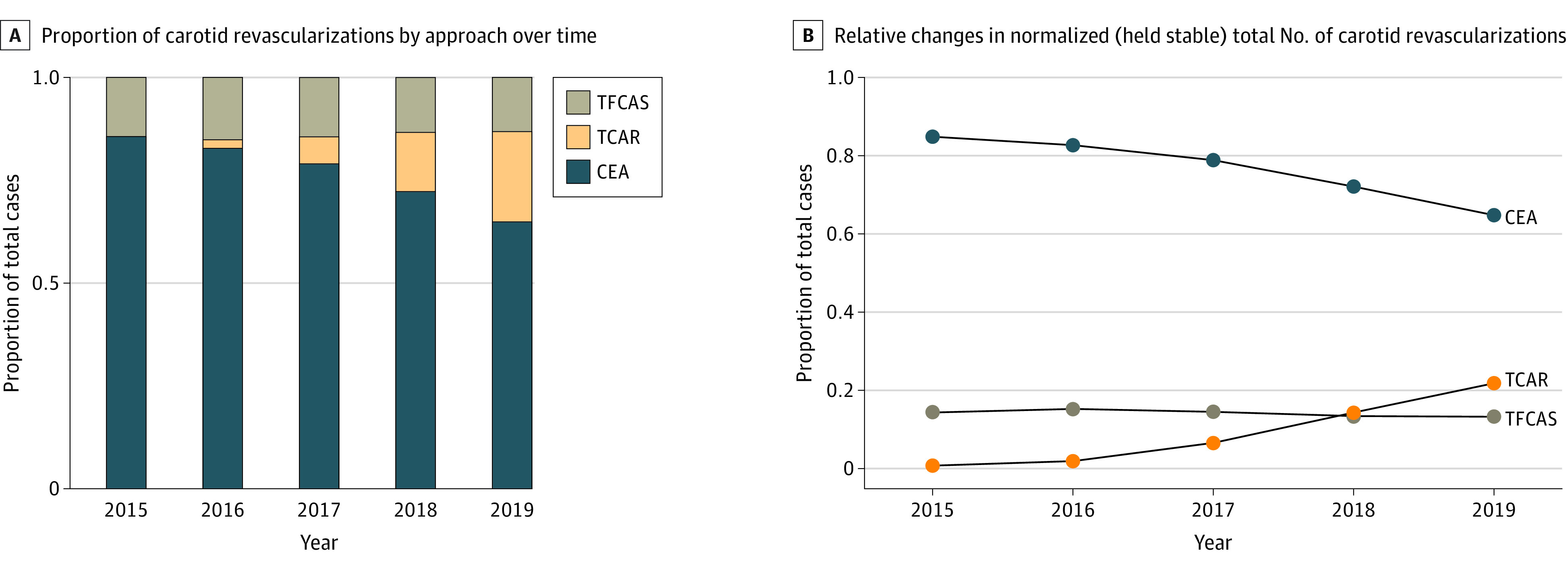
Temporal Trends in Proportional Case Distribution by Operative Approach in the Vascular Quality Initiative, From 2015 to 2019 CEA indicates carotid endarterectomy; TCAR, transcarotid artery revascularization; TFCAS, transfemoral carotid artery stenting.

When carotid revascularization procedures were analyzed by approach as a proportion, the use of CEA decreased over the course of the study period, whereas the use of TFCAS remained stable and TCAR use increased exponentially (Figure 2A). In 2015, CEA was used in 84.9% of all carotid revascularization cases, followed by TFCAS (14.4%) and TCAR (0.8%). In 2018, CEA use decreased to 72.1% of all cases, and TCAR surpassed TFCAS (14.4% vs 13.5% in 2018) as the dominant stenting approach. This pattern persisted and increased through 2019 (CEA, 64.8%; TCAR, 21.9%; TFCAS, 13.3%; *P* < .001). Expressed on a per year basis, the proportional use of CEA decreased by 5.0% (95% CI, −7.4% to −2.6%), TFCAS decreased by 0.3% (95% CI, −1.1% to 0.6%), and TCAR increased by 5.3% (95% CI, 2.3%-8.3%) (Figure 2B).

**Figure 3.**
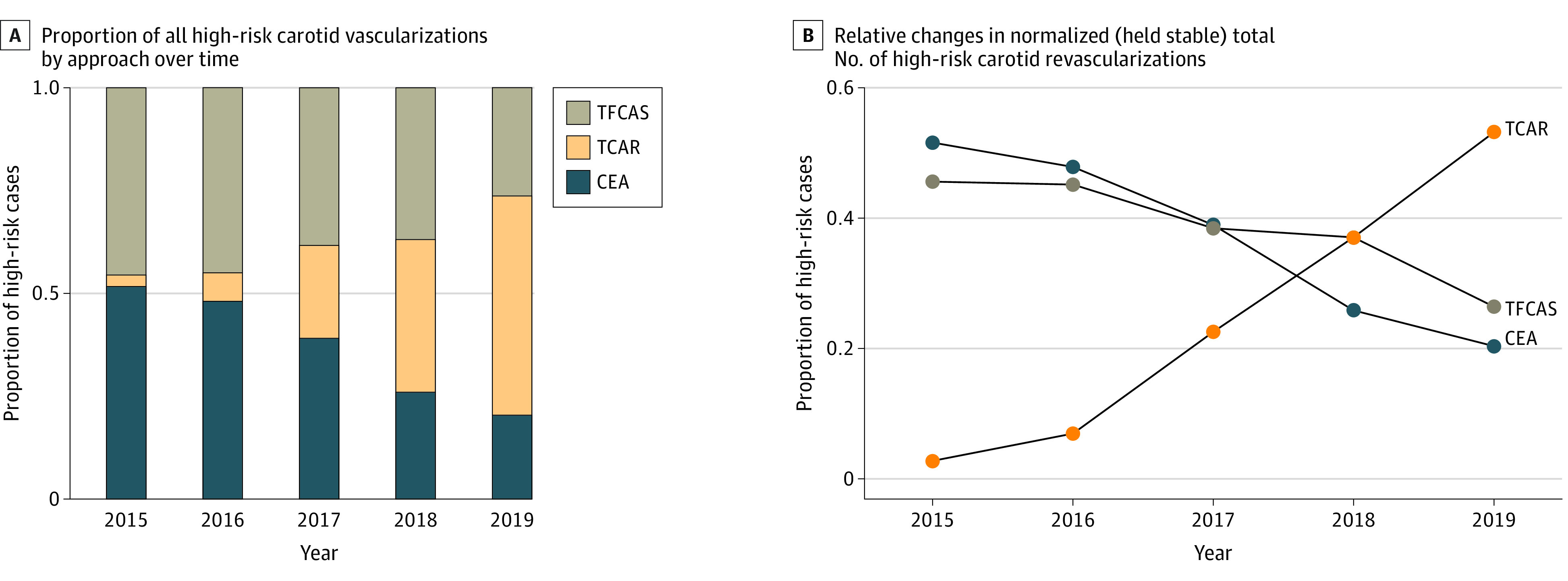
Temporal Trends in Proportional Case Distribution for High-Risk Operative Approach in the Vascular Quality Initiative, From 2015 to 2019 CEA indicates carotid endarterectomy; TCAR, transcarotid artery revascularization; TFCAS, transfemoral carotid artery stenting.

In a sensitivity analysis limited to patients considered to be high risk for the CEA approach on the basis of the Centers for Medicare & Medicaid Services criteria, the temporal changes observed for CEA, TFCAS, and TCAR were greater. In 2015, CEA was used in 51.6% of all carotid revascularization cases, followed by TFCAS (45.6%) and TCAR (2.8%). In 2019, the prevalence of CEA use decreased to 20.3% of cases, TFCAS use decreased to 24.6%, and TCAR use increased significantly to 53.2% (*P* < .001) (Figure 3A). Expressed on a per year basis, the proportional use of CEA for patients with high risk decreased by 7.8% (95% CI, −11.9% to −3.8%), TFCAS decreased by 4.8% (95% CI, −9.5% to −0.14%), and TCAR increased by 12.6% (95% CI, 7.1%-18.1%) (Figure 3B).

### Patient Covariates Associated With Each Operative Approach

Multinomial logistic regression was performed to identify independent patient covariates associated with each operative approach (Table 2). High-risk status was the most important overall characteristic associated with TCAR (RRR, 36.10; 95% CI, 29.24-44.66; *P* < .001) and TFCAS (RRR, 14.10; 95% CI, 11.86-16.66; *P* < .001) vs CEA (reference). Coronary artery disease was the most important individual comorbidity associated with TCAR (RRR, 2.58; 95% CI, 2.31-2.88; *P* < .001) and TFCAS (RRR, 3.99; 95% CI, 3.60-4.43; *P* < .001) compared with CEA. Patients receiving hemodialysis were significantly less likely to undergo TCAR (RRR, 0.21; 95% CI, 0.17-0.26; *P* < .001) and TFCAS (RRR, 0.21; 95% CI, 0.17-0.26; *P* < .001) than CEA. Year of surgery was associated with the likelihood of TCAR (RRR, 2.43; 95% CI, 2.23-2.65; *P* < .001) or TFCAS (RRR, 1.12; 95% CI, 1.08-1.17; *P* = .001) being used, after adjustment for preoperative difference, compared with CEA. Asymptomatic status was associated with decreased odds of TCAR (RRR, 0.59; 95% CI, 0.53-0.66; *P* < .001) vs CEA, but it was not associated with the use of TFCAS vs CEA. A complete summary of all covariates associated with use of TCAR and TFCAS compared with CEA is provided in Table 2.

**Table 2.** Multinomial Logistic Regression to Identify Patient Covariates Associated With Operative Approaches in the VQI Database, From 2015 to 2019

	RRR (95% CI)^a^	*P* value
CEA	1 [Reference]	
**TCAR**		
Age	1.00 (1.00-1.01)	.49
Female sex	1.04 (0.97-1.12)	.23
Race and ethnicity		
Black	1.05 (0.85-1.29)	.68
White	1 [Reference]	
Other^b^	1.03 (0.84-1.25)	.80
Insurance payer		
Medicare	1 [Reference]	
Medicaid	0.81 (0.68-0.96)	.02
Commercial	0.82 (0.71-0.95)	.006
Other or self-pay^c^	1.25 (0.99-1.58)	.07
Hypertension	0.86 (0.77-0.95)	.005
Diabetes	0.83 (0.78-0.88)	<.001
CAD	2.58 (2.31-2.88)	<.001
COPD	0.92 (0.85-0.99)	.27
CHF	0.55 (0.50-0.62)	<.001
CKD or HD	0.21 (0.17-0.26)	<.001
Smoking status	1.02 (0.97-1.06)	.78
Not fully functional status	3.02 (2.58-3.53)	<.001
High-grade stenosis	1.37 (1.23-1.52)	<.001
Asymptomatic status	0.59 (0.53-0.66)	<.001
High-risk status	36.10 (29.24-44.66)	<.001
Year of surgery	2.43 (2.23-2.65)	<.001
**TFCAS**		
Age	0.97 (0.96-0.97)	<.001
Female sex	0.95 (0.95-0.99)	.04
Race and ethnicity		
Black	1.23 (1.04-1.46)	.02
White	1 [Reference]	
Other^b^	0.97 (0.75-1.26)	.84
Insurance payer		
Medicare	1 [Reference]	
Medicaid	1.00 (0.86-1.17)	.97
Commercial	0.94 (0.83-1.05)	.27
Other or self-pay^c^	1.40 (1.18-1.67)	<.001
Hypertension	0.70 (0.64-0.77)	<.001
Diabetes	0.81 (0.77-0.85)	<.001
CAD	3.99 (3.60-4.43)	<.001
COPD	0.90 (0.82-0.98)	.02
CHF	0.54 (0.49-0.60)	<.001
CKD or HD	0.21 (0.17-0.26)	<.001
Smoking status	0.95 (0.92-0.99)	.02
Not fully functional status	3.36 (2.66-4.25)	<.001
High-grade stenosis	1.23 (1.09-1.39)	.001
Asymptomatic status	0.95 (0.84-1.08)	.41
High-risk status	14.10 (11.86-16.66)	<.001
Year of surgery	1.12 (1.08-1.17)	<.001

Abbreviations: CAD, coronary artery disease; CEA, carotid endarterectomy; CHF, congestive heart failure; CKD, chronic kidney disease; COPD, chronic obstructive pulmonary disease; HD, hemodialysis; RRR, relative risk ratio; TCAR, transcarotid artery revascularization; TFCAS, transfemoral carotid artery stenting; VQI, Vascular Quality Initiative.

^a^ Represents the relative risk of undergoing TCAR or TFCAS vs CEA.

^b^ Other race and ethnicity groups were American Indian or Alaska native, Asian, Hispanic, Native Hawaiian or Other Pacific Islander, more than 1 race, and unknown or other.

^c^ Other insurance payers were those that were not Medicare, Medicaid, commercial, or self-pay as coded in the VQI.

A sensitivity analysis repeating this analysis for only patients who underwent carotid revascularization in 2019 was also performed (eTable in the Supplement). Overall, this cohort had similar associations vs the entire cohort, and high-risk status remained associated with the use of TCAR (RRR, 36.20; 95% CI, 28.38-46.05; *P* < .001) and TFCAS (RRR, 18.19; 95% CI, 14.83-22.36; *P* < .001) vs CEA.

### Temporal Changes in Patient Outcomes

Unadjusted MI, stroke, and mortality rates were tabulated across all operative approaches in the VQI database from January 1, 2015 to December 31, 2019 (eFigure 1 in the Supplement). Linear regression revealed no cohortwide association between year of surgery and in-hospital MI, stroke, or mortality.

Centers were dichotomized into a high-volume group or low-volume group, and 45 centers were identified as having performed more than 40 TCAR procedures from 2015 to 2019 after the frequency distribution of TCAR procedures by center was analyzed (eFigure 2 in the Supplement). Overall, in-hospital carotid revascularization outcomes (including TFCAS and CEA) of these high-volume and low-volume centers were compared over the study period. In this cohort-level analysis, there was no association between TCAR volume status and in-hospital outcomes. Specifically, there was no association between year of surgery; TCAR volume status; and mortality, stroke, or MI risk (eFigure 3A in the Supplement). There was also no association detected between frequencies of these in-hospital complications and TCAR volume status when outcomes were analyzed in aggregate (eFigure 3B in the Supplement).

## Discussion

This study found a 13% annual increase in the number of carotid revascularization procedures performed from 2015 to 2019 within the VQI database. In 2018, TCAR surpassed TFCAS as the dominant stenting modality and, in 2019, it was used 65% more often than TFCAS (21.9% vs 13.3%). Even after controlling for patient disease characteristics using multinomial logistic regression, the year of surgery remained associated with the operative approach, suggesting that this change was associated with external (nonpatient) temporal factors. Although the proportion of cases that used TCAR increased each year, patient risk status was the single most important characteristic associated with a stenting approach (ie, TCAR and TFCAS), highlighting the perceived importance of carotid stenting therapies in high-risk patient populations.

The temporal changes in CEA, TCAR, and TFCAS use reported in this study are largely in accordance with the findings in contemporary literature. We observed a higher number of revascularization procedures with time, which is aligned with the increasing age of the US population,^22^ the disproportionate burden of carotid artery stenosis in older adults, and a greater number of institutions subscribing to the VQI.^23^ Because the number of participants in the VQI changes each year, we focused on the proportion of included cases instead of the crude totals. We observed a 5.0% annual decrease in the proportion of carotid revascularization procedures performed with CEA. Multiple studies have illustrated decreasing CEA rates from 1999 to 2015, with 1 study demonstrating a decrease in the national CEA rate from 298 per 100 000 beneficiary-years to 128 per 100 000 beneficiary-years between 1999 and 2014.^24,25^ Findings of the present study represent the continuation of this pattern, with stenting approaches, specifically TCAR, gaining an increasing share of the carotid revascularization landscape over time.

Patient high-risk status was the single most important characteristic associated with TCAR or TFCAS use. This finding likely reflects the current Centers for Medicare & Medicaid Services reimbursement rules on carotid artery stenting, which limited the use of TFCAS and TCAR to patients considered to be high risk for CEA because of certain comorbid or anatomic conditions.^21^ Among carotid revascularization procedures, we found a substantial increase in TCAR use (from 0.8% in 2015 to 21.9% in 2019) and a slight decrease in TFCAS use (14.4% in 2015 to 13.3% in 2019) across the study period. Among patients at high risk, the increased use of TCAR was even greater; by 2019, TCAR represented 53.2% of all carotid revascularization procedures for patients at high risk, increasing from 2.8% in 2015. In addition, TCAR offers the unique benefit of neuroprotection through flow reversal without the need for aortic arch manipulation,^26,27,28,29^ which is often believed to be the main source of atheroembolic events during TFCAS.^30^ In retrospective analyses, TCAR has demonstrated comparable rates of stroke and death but half the risk of in-hospital transient ischemic attack, stroke, and mortality compared with TFCAS.^14,31,32^ A meta-analysis of 30-day mortality rates with TCAR and TFCAS reported that TCAR had lower perioperative stroke and death rates compared with CEA.^33^ However, there are currently no randomized clinical trials comparing outcomes for TCAR vs TFCAS or CEA, and long-term (>1 year) data are lacking. Overall, TCAR has been rapidly adopted by physicians who report data to the VQI because of its favorable safety profile compared with TFCAS, and this approach is becoming important for patients at high risk for stroke, cranial nerve injury, or cardiovascular events. We expect that the use of TCAR will continue to expand now that its application in patients with normal risk was approved by the FDA in May 2022.^8,34^

Chronic kidney disease and hemodialysis were associated with CEA over either stenting approach after adjustment. Patients with CKD or hemodialysis were typically excluded from the major clinical revascularization trials. To our knowledge, the only randomized clinical trial that specifically evaluated the association of CKD with carotid revascularization outcomes in a post hoc analysis was the North American Symptomatic Carotid Endarterectomy Trial, which demonstrated that patients with CKD had similar rates of perioperative stroke and death but higher rates of cardiac events compared with patients with preserved kidney function.^35^ Given that the major benefit from TFCAS is reduced risk of MI,^36,37^ these findings suggest that carotid stenting may be beneficial for patients with CKD. There are no data to support this theory, however. In contrast, data from the Nationwide Inpatient Sample have shown that TFCAS vs CEA is associated with a higher risk of major adverse cardiovascular or cerebrovascular events in patients with CKD,^38^ although these data are based on in-patient observations and do not account for degree of stenosis. In addition, the results from the present analysis suggest that the perceived benefit associated with carotid stenting in patients with CKD is low. It is possible that use of preoperative computed tomography angiography limits the application of TFCAS and TCAR in populations with CKD, as contrast carries a potential risk of contrast-induced acute kidney injury.^39^ It is also possible that patients with CKD have more calcific carotid disease that limits the use of stenting technology. Future analyses of VQI data or similar robust data are necessary to better understand the risks and benefits associated with TFCAS and TCAR for patients with CKD.

The temporal changes in operative approaches that we observed were not associated with overall changes in major in-hospital outcomes. These data are aligned with increasing reports that TCAR performs relatively similarly to CEA.^18,32,40,41,42^ Given that TCAR use increased and CEA use decreased in the 2015 through 2019 period, it is encouraging to see a lack of change in major complications. We attempted to analyze center-level major complications by stratifying according to TCAR use, but we found no difference in outcomes overall or on an annual basis for high-volume or low-volume TCAR centers. A similar analysis evaluating major adverse cardiovascular events after TCAR and CEA found that centers that adopted TCAR had a 10% decrease in the likelihood of major adverse cardiovascular events at 12 months after TCAR adoption compared with centers that continued to perform CEA alone.^40^ The results of the present analysis and previous studies^18,32,40,41,42^ suggest that the introduction of TCAR is associated with stable or slightly improved changes in major in-hospital outcomes after carotid revascularization. Thus, in the future, we expect to see TCAR incorporated into major professional guidelines about the management of extracranial carotid disease.

### Limitations

This study has limitations. The analysis focused on pre–COVID-19 temporal changes to understand practice patterns in a nonpandemic era. The VQI includes a subset of hospitals and surgical centers that choose to participate; thus, this analysis does not represent a true cross-sectional cohort of carotid revascularization practices in the US. The number of carotid revascularization procedures increases year after year, but this increase may be partly attributed to the inclusion of more centers performing carotid revascularization in the study. For this reason, we focused on the proportion of each approach overall and on patients at high risk for stroke, cranial nerve injury, or cardiovascular events, but the general application of the findings across centers that do not participate in the VQI is unclear. In addition, it is impossible the know a surgeon’s rationale for choosing a procedure over another. We evaluated patient covariates that we believed may play a role in this decision, but the analysis may be subject to residual confounding.

There are multiple other studies that report on the outcomes of CEA, TFCAS, and TCAR.^40,43^ We assessed outcomes, including MI, stroke, and mortality, over the study period among all participants in the VQI, and we then dichotomized the centers by high or low volume to investigate the outcomes at centers with early TCAR adoption. Although this analysis did not reveal a detectable association at the cohort level, power was limited by the rarity of these outcomes and was dependent on the number of years in the study, which was small. Future work may include an interrupted time series analysis of the association between choice of operative approach and outcomes given the recent FDA label expansion^44^ and the COVID-19 pandemic, and a longer study period may also be advantageous. We were unable to evaluate cost using VQI data. Recent work by Cui et al^45^ showed that, in symptomatic patients, CEA cost $7821 for 2.85 quality-adjusted life-years and TCAR cost $19 154 for 2.92 quality-adjusted life-years, but by 5 years of follow-up, TCAR had become cost-effective. It is unclear from these data how cost will change over time as TCAR is performed in more asymptomatic patients and as follow-up increases, costs change, and label expansion^44^ and surgeon acceptance lead to further adoption and cost reductions at scale.

## Conclusions

This cohort study showed that since the first TCAR device received FDA approval in 2015, TCAR has overtaken TFCAS as the primary carotid stenting approach in all patients, and TCAR has overtaken TFCAS and CEA as the dominant operative approach in patients at high risk for stroke, cranial nerve injury, or cardiovascular events. The main patient characteristic associated with carotid stenting technologies was high-risk status, whereas CKD was highly associated with CEA. Major in-hospital carotid revascularization outcomes did not significantly change over the study period. Use of TCAR is expected to continue to increase, particularly the expansion of TCAR use in patients with normal risk status.

## Notes

Supplement. **eFigure 1.** MI Rate (%), Stroke Rate (%) and Mortality (%) From 2015 to 2019 Within the VQI **eFigure 2.** Frequency Analysis (A. Histogram, B. Violin Plot) of the Distribution of the Number of TCARs Performed at Each Center From 2015-2019 Within the VQI **eFigure 3.** Yearly Complication Rate (MI, Stroke and Mortality) Stratified Between the High Volume (HV) and Low Volume (LV) TCAR Centers **eTable.** Multinomial Logistic Regression to Identify Patient Covariates Associated With Carotid Revascularization Approach in the VQI for 2019 Only
