# Supplementary material for: Use of Transcarotid Artery Revascularization, Transfemoral Carotid Artery Stenting, and Carotid Endarterectomy in the US From 2015 to 2019
Source: JAMA Netw Open. 2022 Sep 16;5(9):e2231944. doi: 10.1001/jamanetworkopen.2022.31944 (PMC9482062; doi:10.1001/jamanetworkopen.2022.31944)
Supplement: Supplement. — eFigure 1. MI Rate (%), Stroke Rate (%) and Mortality (%) From 2015 to 2019 Within the VQI eFigure 2. Frequency Analysis (A. Histogram, B. Violin Plot) of the Distribution of the Number of TCARs Performed at Each Center From 2015-2019 Within the VQI eFigure 3. Yearly Complication Rate (MI, Stroke and Mortality) Stratified Between the High Volume (HV) and Low Volume (LV) TCAR Centers eTable. Multinomial Logistic Regression to Identify Patient Covariates Associated With Carotid Revascularization Approach in the VQI for 2019 Only [file jamanetwopen-e2231944-s001.pdf]

Supplemental Online Content

Stonko DP, Goldsborough E III, Kibrik P, Zhang G, Holscher CM, Hicks CW. Use of transcarotid artery revascularization, transfemoral carotid artery stenting, and carotid endarterectomy in the US from 2015 to 2019. *JAMA Netw Open*. 2022;5(9):e2231944. doi:10.1001/jamanetworkopen.2022.31944

eFigure 1. MI Rate (%), Stroke Rate (%) and Mortality (%) From 2015 to 2019 Within the VQI

eFigure 2. Frequency Analysis (A. Histogram, B. Violin Plot) of the Distribution of the Number of TCARs Performed at Each Center From 2015-2019 Within the VQI

eFigure 3. Yearly Complication Rate (MI, Stroke and Mortality) Stratified Between the High Volume (HV) and Low Volume (LV) TCAR Centers

eTable. Multinomial Logistic Regression to Identify Patient Covariates Associated With Carotid Revascularization Approach in the VQI for 2019 Only

This supplemental material has been provided by the authors to give readers additional information about their work.

© 2022 Stonko DP et al. *JAMA Network Open.*

eFigure 1. MI Rate (%), Stroke Rate (%) and Mortality (%) From 2015 to 2019 Within the VQI

Unadjusted complication rates over time, with linear regression assessing the associating complication rate with year of surgery. There was no statistically significant association between year of surgery and MI (slope -0.06 %/year [95% CI: -0.09 to 0.02]), stroke (slope -0.03 %/year [95% CI: -0.17 to 0.05]) or in-hospital mortality (slope 0.005 %/year [95% CI: -.02 to 0.03]) over time. Cranial Nerve Injury data was poor early in the carotid artery stenting database so was not pursued here. *MI: myocardial infarction; YR: year.*

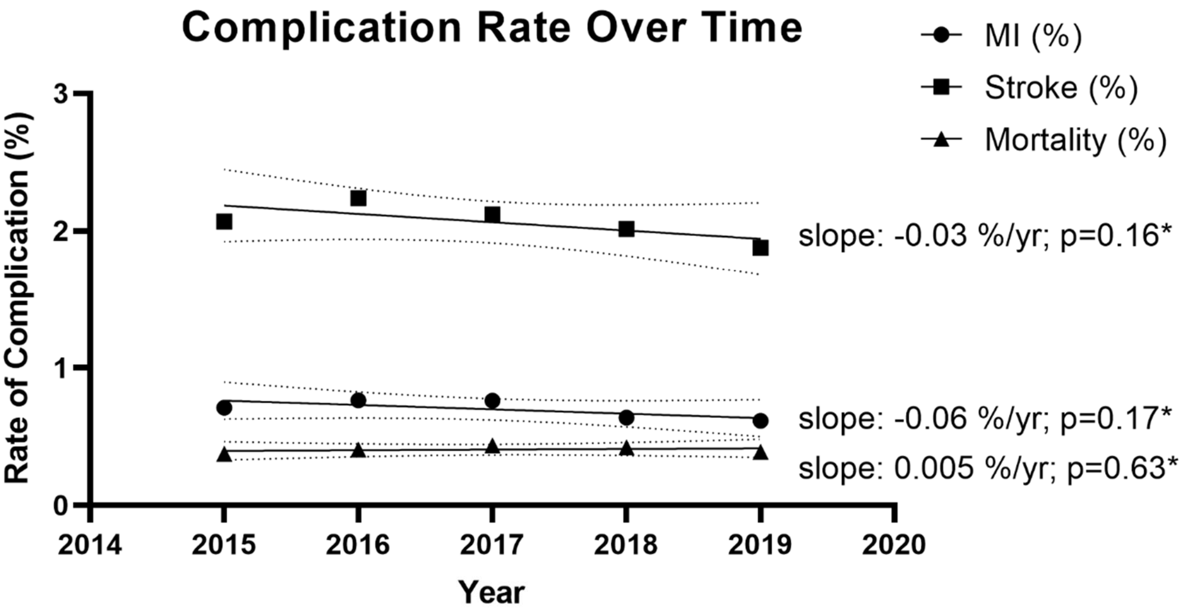

95% CI on Slope:

MI (%)	Stroke (%)	Mortality (%)
-0.08677 to 0.02313	-0.1677 to 0.04666	-0.02259 to 0.03166

*simple linear regression; F test for slope not equal to 0
error ranges represent 95% Confidence Bands

© 2022 Stonko DP et al. *JAMA Network Open.*

eFigure 2. Frequency Analysis (A. Histogram, B. Violin Plot) of the Distribution of the Number of TCARs Performed at Each Center From 2015-2019 Within the VQI

Most centers are performing less than 40 TCARs. Greater than 40 TCARs from 2015-2019 was identified as the high-volume cutoff (n=45), and low volume TCAR centers (n=342). This dichotomization facilitated additional comparison between high-volume and low-volume TCAR centers within this period. *LV: Low Volume; HV: High Volume*

A.

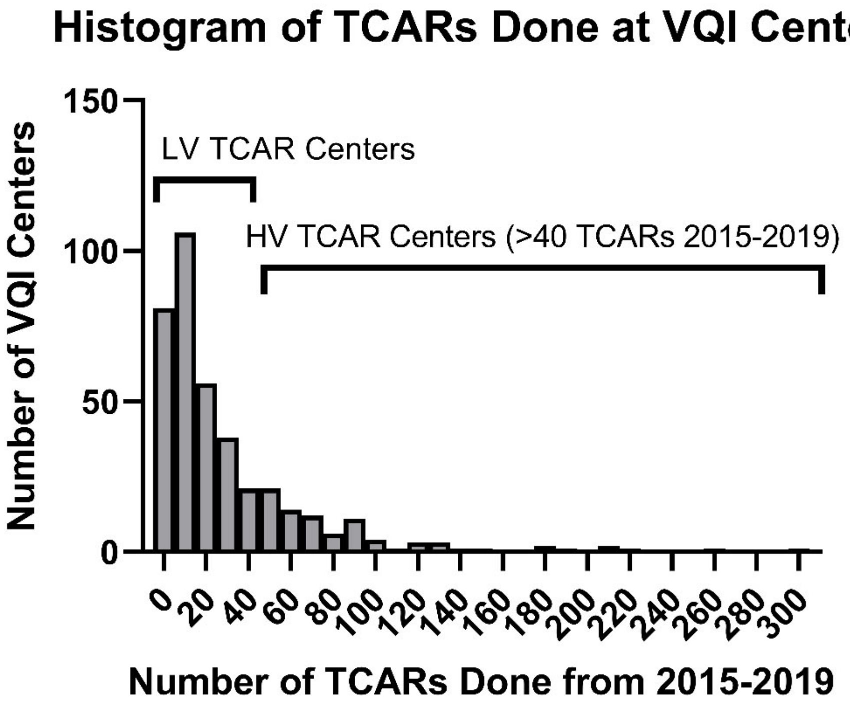

© 2022 Stonko DP et al. *JAMA Network Open.*

B.

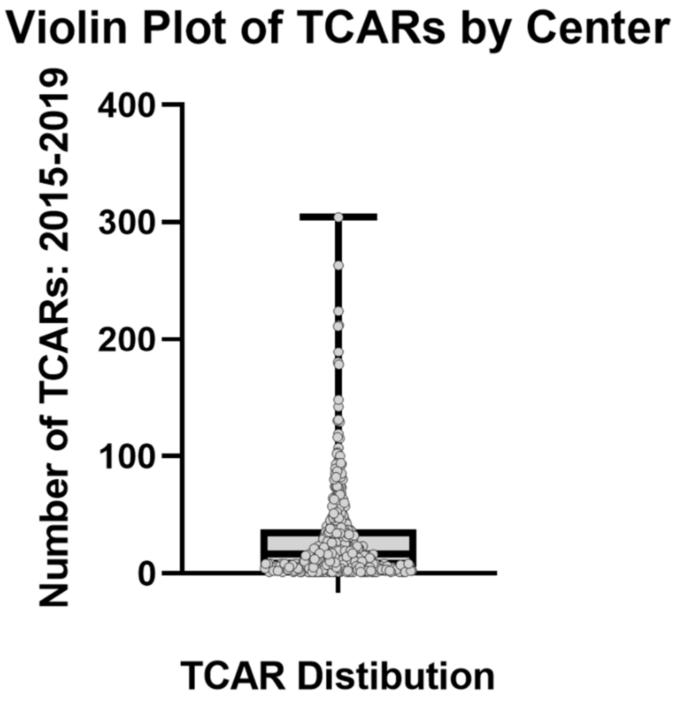

© 2022 Stonko DP et al. *JAMA Network Open.*

eFigure 3. Yearly Complication Rate (MI, Stroke and Mortality) Stratified Between the High Volume (HV) and Low Volume (LV) TCAR Centers

With high and low volume TCAR centers defined as in Supplemental Figure 2, overall outcomes (including TFCAS and CEA) were compared between these center groups over the study period 2015-2019. Overall, in this cohort-wide analysis, there was no statistical association between center TCAR volume status and outcomes. **A.** When accounting for the year of surgery, there was no detectable association between year, center TCAR volume status (high volume or low volume) and mortality (P=0.21), stroke (P=0.28) or MI (P=0.22) risk using t-tests accounting for multiple comparisons. **B.** When year of surgery was removed from consideration. there was also no statistical significance detected between rates of these complications [mortality (P=0.32), stroke (P=0.67) or MI (P=0.52)]. *MI: myocardial infarction, LV: low volume, HV: high volume, SD: standard deviation.*

A.

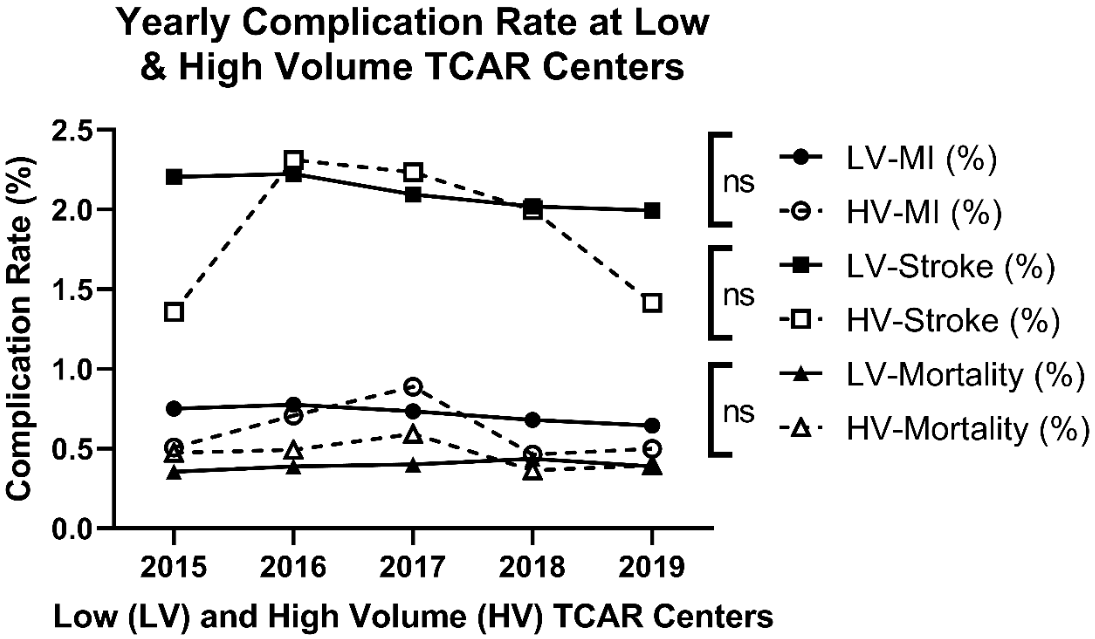

© 2022 Stonko DP et al. *JAMA Network Open.*

B.

Outcomes at Low Volume vs. High Volume TCAR Centers

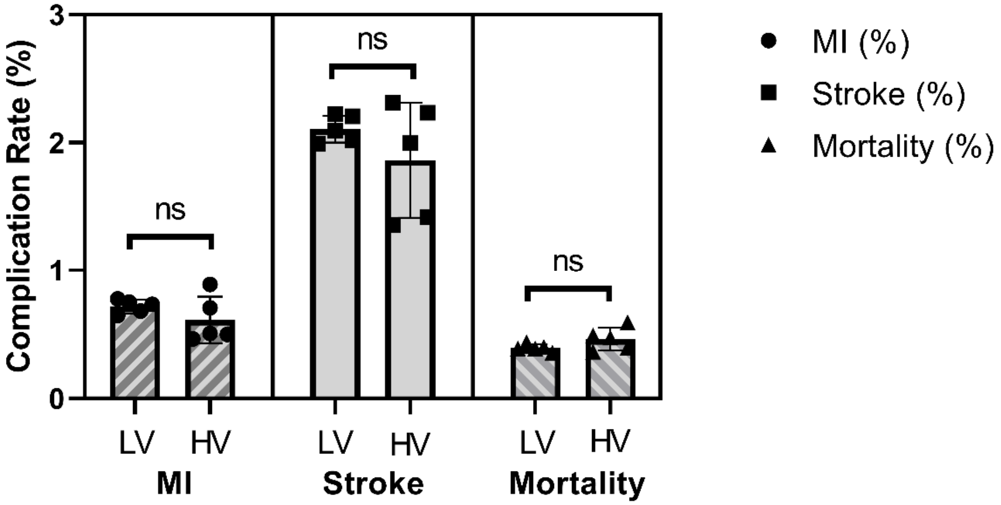

Each point represents the mean complication rate from Low Volume (LV) or High Volume (HV) TCAR centers from one study year, error bars are SD.

© 2022 Stonko DP et al. *JAMA Network Open.*

eTable. Multinomial Logistic Regression to Identify Patient Covariates Associated With Carotid Revascularization Approach in the VQI for 2019 Only

HTN, hypertension; CAD, coronary artery disease; CHF, congestive heart failure; COPD, chronic obstructive pulmonary disease; CKD/HD, chronic kidney disease or hemodialysis.

		Relative Risk Ratio	95 % CI	p
**CEA**	Reference			
**TCAR**				
	Age	1.00	0.99-1.001	0.19
	Female Sex	1.05	0.95-1.16	0.31
	Race (Ref.: White)			
	Black	1.02	0.80-1.30	0.89
	Other	0.92	0.73-1.16	0.50
	Payer (Ref.: Medicare)			
	Medicaid	0.85	0.67-1.08	0.19
	Commercial	0.71	0.60-0.84	<0.001
	Other/Self Pay	1.09	0.81-1.46	0.57
	HTN	0.92	0.80-1.08	0.31
	Diabetes	0.85	0.78-0.93	<0.001
	CAD	2.06	1.81-2.35	<0.001
	COPD	0.90	0.80-0.9998	0.050
	CHF	0.54	0.46-0.63	<0.001
	CKD/HD	0.18	0.13-0.25	<0.001
	Smoking	1.02	0.95-1.09	0.63
	Non-functional Status	3.99	3.25-4.91	<0.001
	High Grade Stenosis	1.37	1.21-1.56	<0.001
	Asymptomatic Status	0.69	0.61-0.78	<0.001
	High Risk Status	36.20	28.4-46.0	<0.001
**TFCAS**				
	Age	0.97	0.96-0.97	<0.001
	Female Sex	0.96	0.87-1.05	0.33
	Race (Ref.: White)			
	Black	1.30	1.02-1.65	0.04
	Other	0.81	0.57-1.15	0.25
	Payer (Ref.: Medicare)			
	Medicaid	1.04	0.82-1.34	0.73
	Commercial	0.85	0.72-1.01	0.06
	Other/Self Pay	1.45	1.10-1.92	0.009
	HTN	0.74	0.64-0.86	<0.001
	Diabetes	0.83	0.75-0.91	<0.001
	CAD	1.94	1.69-2.23	<0.001
	COPD	0.90	0.78-1.02	0.11
	CHF	0.57	0.49-0.66	<0.001

© 2022 Stonko DP et al. *JAMA Network Open.*

	CKD/HD	0.19	0.13-0.29	<0.001
	Smoking	0.98	0.91-1.05	0.57
	Non-functional Status	4.97	3.64-6.81	<0.001
	High Grade Stenosis	1.27	1.10-1.47	0.001
	Asymptomatic Status	1.51	1.27-1.79	<0.001
	High Risk Status	18.19	14.8-22.4	<0.001

© 2022 Stonko DP et al. *JAMA Network Open.*
